# Progress of the Cholera

**Published:** 1847-02

**Authors:** 


*Progress of the Cholera.*—We copy the following from the *Times* newspaper, and we do so at the risk of exciting perhaps unnecessary alarm. It is obvious that the medical profession should not be kept in the dark with respect to such a matter, if for no other reason, for this, that they should be prepared, not only to meet real danger, but to resist and expose any attempt to create premature or uncalled for apprehensions for placehunting or puffing purposes.

"We have received from our correspondent at Trebizonde a letter dated the 26th of September, from which, with deep regret, we make the following extract:—

"The cholera has passed the line of the Russian quarantine on the borders of the Caspian Sea, and is raging throughout all the Tartar villages of the district of Salgau and of Leukeran. A considerable number of Cossacks, from the *cordon* on the Persian frontier, have likewise been attacked. At Rescht, a Persian city in the province of Ghilan, the cholera is still making incessant ravages, which have now continued during two months. The sanatory state of all the towns to the west of the Caspian Sea, from Bakou to Astrachan, is very unfavorable. Dysenteries and diseases of the stomach (frequently mortal) prevail in those towns, particularly amongst the troops in the garrisons. These maladies are probably the forerunners of the real Asiatic cholera—a phenomenon rather curious, which has been again observed latterly in Persia. There prevailed at Teheran, at Astrabad, at Meschid, and at Ispahan, a malady a considerable time before the appearance of the cholera, of which the symptoms resembled the Indian disease. The caravans which arrived from Teheran eight months since spoke of the existence of the scourge which was mistaken for the cholera. A French physician, who resided at Teheran at that period, and who passed through our city a few days since, assured us it was the cholerine, such as had likewise been observed in several towns of Europe in 1833 and 1834* as a precursor of the cholera. The population of Teheran, which had been estimated at 80,000 is reduced by the ravages of the cholera to 60,000. The foreign ministers and their attendants had not dared to return to the city, but still continued to reside at Mount Alburs, in the neighborhood of Schemen, to the north of Teheran. The Russian authorities at Tiflis are well aware of the appearance of the cholera in that neighborhood, and the inhabitants of Tiflis have fled, but up to the 12th of September no official announcement had been made of the fact. Perhaps this course was pursued in order to prevent the merchants from becoming alarmed, and a consequent interruption of commercial affairs."—*Ibid.*

* This is a mistake. The cholera set in in Paris on the 28th of March, 1832.

